# Factors Affecting the Periapical Status of Root-Filled Canals: A Cross-Sectional Study at the Undergraduate Level

**DOI:** 10.1155/2017/7413204

**Published:** 2017-05-21

**Authors:** George Moreira Costa, Suelleng Maria Santos Soares, Paula Cristina Pelli Paiva, Flaviana Dornela Verli, Patrícia Furtado Gonçalves, Sangela Maria da Silva Pereira, Rudys Rodolfo De Jesus Tavarez, Janir Alves Soares, Etevaldo Matos Maia Filho

**Affiliations:** ^1^Department of Dentistry, Federal University of the Vales of Jequitinhonha and Mucuri, Diamantina, MG, Brazil; ^2^Department of Basic Science, Federal University of the Vales of Jequitinhonha and Mucuri, Diamantina, MG, Brazil; ^3^Postgraduate Dentistry, Ceuma University, São Luís, MA, Brazil

## Abstract

**Objective:**

The aim was to evaluate the influence of multiple factors on the periapical status of endodontically treated (ET) teeth.

**Methods:**

The patients were clinically and radiographically reevaluated after root canal therapy. The quality of the root-filled canals, coronal restorations, intraradicular posts, dental caries, and periodontal parameters were associated with the teeth's periapical status.

**Results:**

The 122 patients provided 154 ET teeth; 97.4% teeth were asymptomatic, and 75.5% had a normal periapical status. The percentage of perfect, satisfactory, and deficient root-filled canals was of the order of 41.6%, 46.1%, and 12.3%, respectively. The percentage of adequate and inadequate coronal restorations was 31.2% and 68.8%, respectively. A total of 14.9% teeth had intraradicular posts, and 29.2% had cavitated carious lesions in the dentin. Gingival bleeding was observed in 31.8% of teeth, and dental biofilm was visible in 58.4%. A total of 11.7% showed pathologic tooth mobility, and 22.1% teeth were diagnosed with periodontal disease.

**Conclusions:**

Carious lesions, gingival bleeding, and tooth mobility were significantly associated with the occurrence of periapical lesions in root-filled canals.

## 1. Introduction

Cross-sectional studies aim to evaluate the health status of a population as well as to measure the prevalence of disease or efficacy of the treatments [1]. In therapeutic terms, the oral functions of billions of teeth are preserved through root canal treatment [1, 2]. After shaping, root canal filling, and coronal restoration, the tooth should resume its functions within the oral cavity [3, 4]. At the same time, the previous periapical lesions should be repaired, and this normality should be maintained for a long time [5, 6]. However, no contemporary endodontic treatment protocol ensures 100% clinical and radiographic success [7–10]. In addition, several oral cavity factors may put the success of endodontic treatment at risk [5, 9–11].

In epidemiological terms, the predominance of low technical-quality endodontic treatments is associated with a high prevalence of apical periodontitis [6, 11–13]. Thus, deficient root canal instrumentation would lead to filling failure and, consequently, to the continuity of periapical lesions [11, 13, 14]. Adequate coronal restoration blocks microorganisms and the access of fluids to the root canal in endodontically treated teeth [10]. Coronal-sealing disruption favors microbial recolonization in the root canal and leads to endodontic treatment failure [3, 15]. However, other studies have found no correlation between the quality of coronal restorations and periapical status [14, 16]. With regard to dental caries, an increased risk of developing apical periodontitis has been found in patients with primary carious lesions [10]. Endodontically treated teeth may be compromised by extensive coronary decay and previous periapical lesions [12]. Therefore, the potential of the carious lesions to affect the periapical normality of endodontically treated teeth should be considered [9, 10, 12]. The presence of the intraradicular post [7, 14], distance between the intraradicular post and residual gutta-percha filling, and extension of the residual gutta-percha may influence the periapical status [7, 14, 16].

Retrospective studies have suggested the role that endodontic infections play as local modifying-risk factors to periodontal healing [5, 10]. Similarly, it is necessary to evaluate the influence of periodontal parameters on the periapical status of endodontically treated teeth [17]. It is worth highlighting that many conclusions are based solely on radiographic interpretations [3, 12, 13, 18]. Thus, the current study aims to clinically and radiographically assess the influence of the quality of root canal fillings, including the type, extent, and quality of coronal restorations; presence of intraradicular posts; space between the post and filling and the extension of the remaining apical gutta-percha; type, involvement, cavitation of carious lesions, and several clinical periodontal parameters on the periapical status of the root-filled canals undergraduate students have accomplished.

## 2. Material and Methods

The current research was conducted in full compliance with the ethical principles stated in the Declaration of Helsinki and was approved by the Ethics Committee of Research (Protocol number 061/09). The patients included in this study required root canal therapy in teeth with only one or two root canals and were attended by undergraduate students in their first clinical experience in the area of endodontics. Such students were in the 3rd undergraduate year and had prior experience in laboratory training using anterior and premolars teeth extracted from humans. Therefore, patient with calcified root canals and with excessive root curvatures or retreatment cases and molar teeth were excluded from this stage clinic. The clinical appointments were performed at the Endodontics Clinic of UFVJM between February 2006 and December 2010. The patients were invited to clinical and radiographic recall 1 to 6 years after the completion of the root canal therapy. They signed informed consent forms. Previously calibrated examiners used structured forms and obtained clinical and radiographic data describing the endodontically treated teeth.

### 2.1. Endodontic Treatment Protocol

The endodontic treatment followed a defined protocol [14]. After the absolute isolation of the tooth with a rubber dam, followed by operative field antisepsis with 5% iodinated alcohol and 3% hydrogen peroxide, coronal access was accomplished. The root canals were explored with #6 to #15 K-files (Maillefer Instruments, Ballaigues, Switzerland) and prepared by the manual crown-down concept using K-files and Gates-Glidden drills (Maillefer Instruments, Ballaigues, Switzerland). The root canals were irrigated with 1%, 2.5%, or 5.25% sodium hypochlorite solutions (Biodinâmica Laboratórios, São Paulo, Brazil) for cases such as vital, necrotic, or necrosis pulp associated with periapical lesions, respectively. Irrigation was performed using a Luer Lock syringe with a 27 G needle. The WL was set at 1.0 mm from the radiographic apex. Apical patency was obtained only in root canals with necrotic pulp. The apical segment of the root canal was shaped using the step-back technique until the preflaring limit was reached. After smear layer removal using 17% EDTA solution and final irrigation with NaOCl solution, the root canals were dried with paper points. The main gutta-percha cone (Odous, Belo Horizonte, MG, Brazil) was set to the WL, and the canal was filled according to the thermomechanical technique using an epoxy resin-based endodontic sealer (Sealer 26, Dentsply, Petrópolis, RJ, Brazil). After coronal restoration was completed, a final radiograph of the root filling was taken in accordance with the bisecting-angle technique and processed following the time-temperature method.

### 2.2. Calibrating the Examiners

Prior to the research, a graduate student clinically assessed the quality of coronal restorations and endodontic-origin signs and symptoms of 20 patients, and he recorded several clinical periodontal parameters. Substantial* kappa* agreement level was obtained (*κ* > 0.8). Next, periapical radiographs of 20 endodontic treatments performed in patients who did not participate in the current study were obtained from the integrated clinic records. These radiographs were used to calibrate the three examiners so they could classify the quality of the root canal fillings, restorations, and periapical status of the respective teeth. There were intraexaminer and interexaminer agreement of 0.82–0.92 and 0.76–0.80, respectively.

### 2.3. Clinical and Radiographic Assessment

Artificial lighting was used on the teeth in relative isolation. The analyzed clinical signs and symptoms were edema, erythema, fistula, purulent exudate drainage, and pain on palpation of the adjacent mucosa in addition to the vertical percussion of the endodontically treated tooth. Carious lesions were classified according to type (primary or secondary), degree of involvement of the mineralized structures (enamel, dentin, and cementum), and cavitation (present or absent).

The coronal restorations of endodontically treated teeth were clinically assessed using exploratory probe 5 (Duflex, São Paulo, SP, Brazil). The restorations were classified as I: adequate when restoration was achieved with a permanent resin-based composite material, amalgam, or artificial prosthetic crown (marginal sealing was considered adequate when there was no probe retention or when no dental caries were detected) and II: inadequate when restoration was achieved with temporary materials (glass ionomer, zinc oxide eugenol, or zinc phosphate cements); when the restorations showed excess material in the cervical region, cracks, dental caries, perforations, open margins, or fracture; or when the restorations were absent.

Periodontal probing was performed on buccal, lingual, mesial, and distal surfaces using a millimeter periodontal probe (Williams, Golgran, São Paulo, SP, Brazil). Six sites per tooth were assessed, and the greater measurement value for each surface was recorded. Visible bacterial biofilm, gingival bleeding on probing, and pathologic tooth mobility were classified as being present or absent. Periodontal disease was considered to be present when the tooth simultaneously presented with one or more sites with a pocket depth ≥ 4 mm and an insertion loss ≥ 3 mm.

A periapical radiograph of each patient was performed using ultraspeed film (Eastman Kodak Co., Rochester, NY) according to the bisector technique and aided by an intraoral positioner (Maquira Indústria de Produtos Odontológicos Ltda, Maringá, PR, Brazil). Three previously calibrated examiners assessed these radiographs and rated the quality of the coronal restorations, quality of the root canal fillings, and periapical status. Using a projector (Kodak, Ektagraphic Universal Slide Tray, Kodak Company, NY, USA), the radiographs were projected in a dark room with 6x magnification and individually assessed by three calibrated examiners.

The coronal restorations were classified as I: adequate when exhibiting a well-adapted restoration in the cervical region of the proximal surfaces and II: inadequate when exhibiting overcontour signs on the proximal surfaces, open margins, or recurrent carious lesions. The clinical and radiographic findings were associated with the final classification of the restorations.

**Figure 1:**
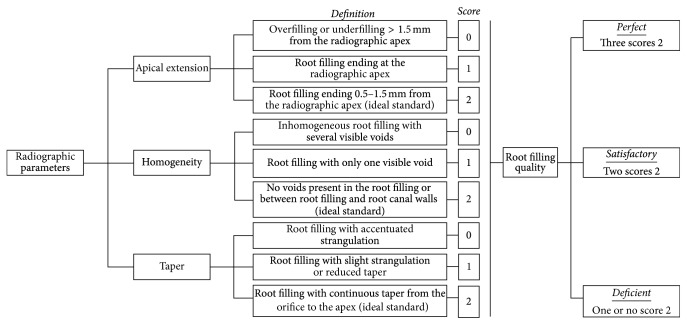
Drawing of the root filling quality standard as a function of radiographic parameters [14].

The radiographic quality of the fillings was classified according to the apical extension, homogeneity, and taper [14] by considering the tooth as the sample unit. When the tooth had more than one root canal, the worst-quality root canal was considered. The apical edge was measured using an image projected in a grid pattern. The apical limit, homogeneity, and taper were subclassified in scores: 0 (accentuated deviation from normality), 1 (mild deviation from normality), and 2 (gold standard). Data on the external root morphology and on the initial root canal diameter were considered to reduce biases when the taper parameter was assessed [14]. The frequency of scores determined whether the filling quality was perfect, satisfactory, or deficient (Figure 1).

The periapical status was classified as I: normal when there was no periapical radiolucency or when the mild thickening of the apical periodontal ligament did not exceed twice the lateral periodontal ligament thickness and II: altered when there was defined periapical radiolucency in connection with the apical portion of the root, with a thickness twice as great as the lateral periodontal ligament width [14].

The intraradicular posts were evaluated for the extent of the remaining gutta-percha and for the existence of a void between the filling material and post.

### 2.4. Endodontic Treatment Success Criteria

The treatment was considered successful when the periapical radiographic normality was associated with the absence of clinical signs and symptoms. The presence of periapical radiolucency was defined as treatment failure.

### 2.5. Statistical Analysis

The descriptive data analysis was conducted by determining the frequency distribution. Pearson's chi-squared tests, Fisher's exact test, and bivariate and multivariate logistic regression analyses were used to identify possible associations between the independent variables and the clinical and radiographic success of the endodontically treated teeth. The significance level was set at 5%.

## 3. Results

The sample comprised 122 patients aged between 16 and 60 years (39.35 ± 11.56). One hundred and fourteen (74.0%) of the 154 evaluated teeth were single-rooted, and 20 were two-rooted premolars. The periapical region had a normal radiographic appearance in 116 (75.3%) teeth. Four patients (3.3%) presented with periapical pathology, 3 had chronic periapical abscesses, and 1 had acute apical periodontitis. Clinical and radiographic success was found in 113 (73.4%) teeth. Gender, age group, teeth group, and the presence of adjacent or antagonist teeth did not significantly influence the clinical or radiographic success of the treatment (Table 1).

The prevalence of teeth with perfect, satisfactory, and deficient root-filled canals was 41.6%, 46.1%, and 12.3%, respectively. The quality standard of the fillings did not vary according to the tooth group (*p* = .904), and the quality of the fillings did not influence the clinical or radiographic success (*p* = .751). There was a high prevalence of gold-standard scores in the three filling parameters (*p* < .05); however, no parameter significantly affected treatment success (*p* > .05) (Table 2).

Table 3 presents the clinical and radiographic successes according to the coronal restoration parameters. Of the 129 (83.8%) teeth that were restored, 81 (62.8%) were improperly restored, and 101 (78.3) had permanent-type material. Seventy-nine (61.2%) teeth had restorations with more than two surfaces, and 78 (60.5%) teeth had intracoronal restoration. Only 23 (14.9%) teeth had intraradicular posts; 15 (9.8%) of these had a void between the remaining gutta-percha and posts, but the remaining apical gutta-percha was smaller than a 4 mm extension in only 5 (3.2%) teeth. None of these factors significantly influenced endodontic treatment success (*p* > .05).

Carious lesions were identified in 45 (29.2%) teeth and were predominantly secondary (82.2%), located on the dentin (75.5%), and had cavitation (60%). The presence of carious lesions significantly influenced treatment success (*p* = .005) (Table 4).

Regarding the periodontal parameters, 90 (58.4%) teeth showed visible bacterial biofilm deposits, which did not significantly influence the periapical status. Gingival bleeding on probing was found in 49 (31.8%) teeth and was associated with a significantly low success rate (*p* = .020). Pathological tooth mobility was found in 18 (11.7%) teeth, and it significantly influenced treatment success (*p* = .017). However, periodontal disease was identified in 34 (22%) teeth but did not influence periapical status (Table 5). The multivariate logistic regression analysis showed that carious lesions, gingival bleeding on probing, and tooth mobility were significantly associated with failed endodontic treatment (Table 6). Therefore, the null hypothesis was rejected.

## 4. Discussion

Following careful analysis of multiple clinical and radiographic factors, we found an endodontic treatment success rate of 73.4%. This percentage was higher than that found in previous studies [6, 11, 13, 19]. These results may be interpreted as a function of the patient demographic characteristics, presence of adjacent and antagonist teeth to the endodontically treated tooth, quality of the root-filled canal, quality of the coronal restorations, presence of intraradicular posts, operator experience, caries, and periodontal alterations [16, 19–21].

The sampling consisted of 114 single-rooted teeth and 20 two-rooted premolars. However, no significant difference was found between the teeth and the periapical status, a fact that corroborates the findings of Ng et al. [1] study. Comparatively, in other clinical studies, there was a lower prevalence of periapical lesions in incisors and canine teeth in relation to premolars and molars [19, 20]. However, the some studies have reported higher periapical lesions rates among incisors [4, 21, 22].

Gender, age group, antagonist, and adjacent teeth did not significantly affect the success of the endodontically treated teeth. Benenati and Khajotia [8] conducted a study on treatments performed by undergraduate students and found that patient, gender, and age group did not influence their endodontic success. Farzaneh et al. [9] and Moradi and Gharechahi [21] highlighted the influence of gender on the periapical status of endodontically treated teeth. In addition, they concluded that age should not be considered a risk factor. Nevertheless, aging may contribute to endodontic treatment failure [23]. The healing process in older patients is presumably slower and not as effective due to the physiological aging process [5]. In contrast, Matsumoto et al. [7] found that the lack of at least one adjacent tooth favors treatment failure.

In their first clinical experience, undergraduate students obtained a high prevalence of gold-standard scores in three filling quality parameters. Most filling quality assessments are based on apical limit and homogeneity parameters [7, 8, 12, 19, 21, 23], and few studies assess taper [14, 20]. Deficient root canal taper may favor the persistence of endodontic posttreatment periapical lesions [11, 14]. The current study used a strict radiographic evaluation methodology to assess the fillings; however, the high-quality technical standard did not significantly influence the endodontic treatment success.

As for coronal restorations, only 37.2% were of adequate quality, and 16.2% of the teeth had no coronal restorations. Despite this low quality standard, the restoration factor did not influence treatment success. However, Ray and Trope [15] reported a significant correlation between the adequate quality of coronal restoration and periapical normality. Oppositely the coronal restoration showed no association with the presence of apical periodontitis when the root canals were properly filled [11, 17, 19]. The coronal-leakage problem may not be of great clinical importance, as several in vitro studies suggest [11, 20]. Well-prepared and filled root canals resist bacterial penetration, even upon direct and long-standing oral exposure by dental caries, fractures, or restoration loss [16, 19]. In this aspect Tronstad et al. [17] attested that the technical quality of the endodontic treatment as judged radiographically was significantly more important than the technical quality of the coronal restoration when the periapical status of endodontically treated teeth was evaluated. Overall, the chance of apical periodontitis healing increases when both appropriate endodontic and restorative treatments are performed [3, 24]. Therefore, immediate permanent restoration is highly recommended due to its specific functions and to the fact that permanent restoration is a cofactor of the endodontically treated tooth success and longevity [24, 25].

Intraradicular posts do not influence endodontic treatment success [16]. The presence of intraradicular posts and a void between them and the filling did not influence periapical status in the current study. Nevertheless we speculated that this empty space could favor the proliferation of microorganisms and consequently establish a periapical lesion. Based on the results obtained in this study, the hypothesis was rejected. However, it is worth emphasizing that most fillings (78.2%) had remaining gutta-percha greater than or equal to 4 mm, which was enough to seal the root canal [7, 8]. Corroborating this statement, a higher prevalence of periapical lesions was observed in teeth with intraradicular posts with less than 4 mm of remaining gutta-percha [14]. Therefore, other clinical studies could ascertain the influence of these voids in the periapical state of the teeth with adequate remaining gutta percha apical.

The carious lesions in the current study were negative prognostic factors of endodontic treatment success. Marginal decay did not influence periapical status [19]. However, it is plausible that the microorganisms found in the depth at which dentin caries cavities reach the pulp chamber via dentinal tubules colonize the root canal system and induce the formation or maintenance of periapical lesions. Chen et al. [12] correlated dental-caries severity with the occurrence of periapical lesions. In their study, endodontically treated teeth with carious lesions extending up to the pulp chamber had a high prevalence of periapical lesions. Thus, the effective control of dental caries prior to root canal treatment [5] should also be maintained after endodontic treatment completion to prevent the development of caries and prevent microorganisms from accessing the root canals and periapical tissues.

The current study found that the bleeding on probing and pathologic tooth mobility were predictors of endodontic treatment failure. Studies that evaluated periodontal aspects related to periapical status are mainly based on the periodontal pocket depth and marginal periodontium-insertion loss [5, 7, 12]. Periodontal bone support reduction has a negative effect on endodontic treatment prognosis [10, 12], but few studies have linked periodontal disease to pulp [22, 26] and periapical diseases [5]. It has been suggested that occlusal trauma is associated with an increased chance of unfavorable periapical healing. Moreover, the severity of marginal bone loss was positively correlated with the number and size of periapical radiolucencies [12].

Therefore, gingival bleeding and increased tooth mobility are common periodontium inflammatory diseases that can occur before or after a root canal treatment, and these diseases could delay or jeopardize periapical healing. Because periodontal disease was found in 22% of the endodontically treated teeth, although it did not significantly affect periapical status, it is conjectured that periodontal pathology negatively affects the periapical region via the periodontal ligament, only in its most advanced stage [26]. Perhaps in endodontically treated teeth devoid of immune-defense mechanisms conferred by vital pulp tissue, the microorganisms of periodontal disease can colonize the pulp cavity following the path of lateral canals and dentinal tubules and alter normal periapical tissues. From this perspective, endodontically treated teeth require combined attention focused on controlling periodontal disease [6] and coronary shielding [16, 26] because they are important predictors of periapical normality and the longevity of teeth with adequate endodontic treatments.

In addition, it is worth highlighting the limitations of cross-sectional clinical studies in comparison to longitudinal studies. The clinical data record related to caries, the quality of coronary restorations, periodontal clinical parameters, and all specific endodontic in formation at the baseline phase could provide valuable scientific evidence. Another limitation of the current study was the poor adherence of the patients to the recalls. Obviously controlled clinical studies should be performed to validate the influence of periodontal changes on the periapical state of endodontically treated teeth.

The root canal fillings of endodontic treatments performed by undergraduate students were of high quality. Advanced carious lesions, gingival bleeding, and abnormal tooth mobility were significantly associated with the occurrence of periapical lesions.

## Figures and Tables

**Table 1:** Clinical and radiographic success according to demographic variables.

Variables	*n*	Clinical and radiographic results				*p* ^*∗*^
		Success		Failure		
		*n*	%	*n*	%	
Gender						
Male	40	30	75.0	10	25.0	0.787
Female	114	83	72.8	31	27.2	
Age group (years)						
16–25	22	16	72.7	6	27.3	0.142
26–35	35	21	60.0	14	40.0	
36–45	50	37	74.0	13	26.0	
46–60	47	39	83.0	8	17.0	
Teeth group						
Single-rooted	113	80	70.8	33	29.2	0.229
Two-rooted	41	33	80.5	8	19.5	
Adjacent tooth						
Present	124	91	73.4	33	26.6	0.995
Absent	30	22	73.3	8	26.7	
Antagonist tooth						
Present	141	104	73.8	37	26.2	0.747
Absent	13	9	69.2	4	30.8	

^*∗*^Pearson's chi-square test and Fisher's exact test.

**Table 2:** Clinical and radiographic success according to filling quality parameters.

Variables	*n*	Clinical and radiographic results				*p* ^*∗*^
		Success		Failure		
		*n*	%	*n*	%	
Apical extension						
0	33	23	69.7	10	30.3	0.239
1	8	4	50.0	4	50.0	
2	113	86	76.1	27	23.9	
Homogeneity						
0	13	9	69.2	4	30.8	0.830
1	15	12	80.0	3	20.0	
2	126	92	73.0	34	27.0	
Taper						
0	19	15	78.9	4	21.1	0.581
1	22	18	81.8	4	18.2	
2	113	80	70.8	33	29.2	
Quality of fillings						
Perfect	64	46	71.9	18	28.1	0.751
Satisfactory	71	54	76.1	17	23.9	
Deficient	19	13	68.4	6	31.6	

^*∗*^Pearson's chi-square test and Fisher's exact test.

**Table 3:** Clinical and radiographic success according to coronal restoration parameters.

Variables	*n*	Clinical and radiographic results				*p* ^*∗*^
		Success		Failure		
		*n*	%	*n*	%	
Occurrence						
Present	129	97	75.2	32	24.8	0.246
Absent	25	16	64.0	9	36.0	
Quality of the restorations						
Adequate	48	37	77.1	11	22.9	0.702
Inadequate	81	60	74.1	21	25.9	
Type						
Permanent	101	75	74.3	26	25.7	0.640
Temporary	28	22	78.6	6	21.4	
Number of surfaces						
≤2 surfaces	50	38	76.0	12	24.0	0.866
>2 surfaces	79	59	74.7	20	25.3	
Extension						
Intracoronal	78	57	73.1	21	26.9	0.488
Onlay	25	18	72.0	7	28.0	
Total crown	26	22	84.6	4	15.4	
Intra-radicular post						
Present	23	19	82.6	4	17.4	0.320
Absent	131	94	71.8	37	28.2	
Void between post and gutta-percha						
Present	15	13	86.7	2	13.3	0.589
Absent	8	6	75.0	2	25.0	
Remaining gutta-percha						
≥4 mm	18	16	88.9	2	11.1	0.194
<4 mm	5	3	60.0	2	40.0	

^*∗*^Pearson's chi-square test and Fisher's exact test.

**Table 4:** Clinical and radiographic success according to carious lesion parameters.

Variables	*n*	Clinical and radiographic results				*p* ^*∗*^
		Success		Failure		
		*n*	%	*n*	%	
Occurrence						
Present	45	26	57.8	19	42.2	**0.005**
Absent	109	87	79.8	22	20.2	
Type						
Primary	8	5	62.5	3	37.5	1.000
Secondary	37	21	56.8	16	43.2	
Involvement						
Enamel	7	4	57.1	3	42.9	0.880
Dentin	34	19	55.9	15	44.1	
Cementum	4	3	75.0	1	25.0	
Cavitation						
Present	27	15	55.6	12	44.4	0.712
Absent	18	11	61.1	7	38.9	

^*∗*^Pearson's chi-square test and Fisher's exact test.

**Table 5:** Clinical and radiographic success according to periodontal parameters.

Variables	*n*	Clinical and radiographic results				*p* ^*∗*^
		Success		Failure		
		*n*	%	*n*	%	
Visible biofilm						
Present	90	64	71.1	26	28.9	0.451
Absent	64	49	76.6	15	23.4	
Bleeding on probing						
Present	49	30	61.2	19	38.8	**0.020**
Absent	105	83	79.0	22	21.0	
Tooth mobility						
Present	18	9	50.0	9	50.0	**0.017**
Absent	136	104	76.5	32	23.5	
Periodontal disease						
Present	34	26	76.5	8	23.5	0.644
Absent	120	87	72.5	33	27.5	

^*∗*^Pearson's chi-square test and Fisher's exact test.

**Table 6:** Bivariate and multivariate logistic regression between independent variables and treatment failure (*n* = 154).

Independent variables	Level	*n*	Bivariate analysis			Multivariate analysis		
			OR	CI 95%	*p*	OR	CI 95%	*p*
Teeth group	Single-rooted (reference)	113	1.00	—	—	1.00	—	—
	Two-rooted	41	0.58	0.24–1.40	0.232	0.54	0.20–1.43	0.221

Quality of root-filled canal	“Perfect” (reference)	64	1.00	—	—	1.00	—	—
	Satisfactory	71	0.80	0.37–1.73	0.580	0.69	0.29–1.64	0.405
	Poor	19	1.08	0.62–1.89	0.771	1.31	0.67–2.53	0.420

Quality of coronal restorations	Adequate (reference)	48	1.00	—	—	1.00	—	—
	Inadequate	81	1.17	0.51–2.71	0.702	0.80	0.29–2.19	0.668

Carious lesion	Absent (reference)	109	1.00	—	—	1.00	—	—
	Present	45	2.89	1.35–6.14	**0.006** ^**∗**^	2.97	1.21–7.28	**0.017** ^**∗**^

Bleeding on probing	Absent (reference)	105	1.00	—	—	1.00	—	—
	Present	49	2.38	1.13–5.02	**0.021** ^**∗**^	2.45	1.02–5.84	**0.043** ^**∗**^

Tooth mobility	Absent (reference)	136	1.00	—	—	1.00	—	—
	Present	18	3.25	1.18–8.88	**0.022** ^**∗**^	4.23	1.23–14.51	**0.022** ^**∗**^

Periodontal disease	Absent (reference)	120	1.00	—	—	1.00	—	—
	Present	34	0.81	0.33–1.97	0.644	0.34	0.10–1.11	0.076

*Note*. Statistical significance; OR, *Odds Ratio*; CI, confidence interval.
